# Lost in transition: A protocol for a retrospective, longitudinal cohort study for addressing challenges in opioid treatment for transition-age adults

**DOI:** 10.1371/journal.pone.0297567

**Published:** 2024-08-14

**Authors:** Josh Aleksanyan, Sugy Choi, Patricia Lincourt, Constance Burke, Kelly S. Ramsey, Shazia Hussain, Ashly E. Jordan, Maria Morris, Thomas D’Aunno, Sherry Glied, Jennifer McNeely, Brian Elbel, Tod Mijanovich, Samrachana Adhikari, Charles J. Neighbors

**Affiliations:** 1 Department of Population Health, New York University Grossman School of Medicine, New York, New York, United States of America; 2 New York State Office of Addiction Services and Supports (OASAS), Albany, New York, United States of America; 3 New York University Wagner School of Public Policy, New York, New York, United States of America; 4 Department of Applied Statistics, Social Science, and Humanities, New York University Steinhardt School of Culture, Education, and Human Development, New York, New York, United States of America; PLOS: Public Library of Science, UNITED KINGDOM OF GREAT BRITAIN AND NORTHERN IRELAND

## Abstract

**Background:**

In the United States, there has been a concerning rise in the prevalence of opioid use disorders (OUD) among transition-age (TA) adults, 18 to 25-years old, with a disproportionate impact on individuals and families covered by Medicaid. Of equal concern, the treatment system continues to underperform for many young people, emphasizing the need to address the treatment challenges faced by this vulnerable population at a pivotal juncture in their life course. Pharmacotherapy is the most effective treatment for OUD, yet notably, observational studies reveal gaps in the receipt of and retention in medications for opioid use disorder (MOUD), resulting in poor outcomes for many TA adults in treatment. Few current studies on OUD treatment quality explicitly consider the influence of individual, organizational, and contextual factors, especially for young people whose social roles and institutional ties remain in flux.

**Methods:**

We introduce a retrospective, longitudinal cohort design to study treatment quality practices and outcomes among approximately 65,000 TA adults entering treatment for OUD between 2012 and 2025 in New York. We propose to combine data from multiple sources, including Medicaid claims and encounter data and a state registry of substance use disorder (SUD) treatment episodes, to examine three aspects of OUD treatment quality: 1) MOUD use, including MOUD option (e.g., buprenorphine, methadone, or extended-release [XR] naltrexone); 2) adherence to pharmacotherapy and retention in treatment; and 3) adverse events (e.g., overdoses). Using rigorous analytical methods, we will provide insights into how variation in treatment practices and outcomes are structured more broadly by multilevel processes related to communities, treatment programs, and characteristics of the patient, as well as their complex interplay.

**Discussion:**

Our findings will inform clinical decision making by patients and providers as well as public health responses to the rising number of young adults seeking treatment for OUD amidst the opioid and polysubstance overdose crisis in the U.S.

## Introduction

Transition-age (TA) adulthood—18 to 25-years old—is a distinct developmental phase during which a person’s biological, psychological, and sociological aspects are evolving [1–4]. TA adults differ from adolescents in that their brain development is further progressed and their social roles allow for more independence. However, TA adults differ from older adults in that their neurocognitive development is not complete, and their social roles and institutional ties remain in flux. TA adults undergo consequential shifts in their circumstances. They experience greater independence from caregivers and childhood support systems, face increasing societal expectations of self-sufficiency, and continue self-exploration and experimentation, as they are yet to take on full adult responsibilities related to lifelong roles in occupations, partnerships, and as caregivers [2–6].

During this critical life phase, TA adults are more likely to use substances and have higher prevalence of substance use disorder (SUD) than adults aged 26 or older (14.4% vs. 7.2% in 2019). Paradoxically, they are less likely to receive specialty treatment for SUD than adults aged 26 or older (7.4% vs. 14.2% in 2019) [7]. Not only is TA adulthood a time of increased risk of developing SUD but also a time when prolonged exposure to substances can have lasting effects on brain development and future role functioning [8, 9]. Brain development extends into the mid-20s, during which time neurological areas associated with goal-seeking, emotional processing, and self-regulation continue to mature. The brain reward system is more developed than the inhibitory system among TA adults, contributing to potentially more harmful substance use and addiction [10–12].

Currently, the United States is experiencing an epidemic of opioid use disorders (OUD), including an opioid and polysubstance overdose crisis, increasingly among TA adults [13–15]. OUD are chronic conditions that require long-term treatment engagement for the best prognosis [16–20]. Individuals with OUD have high mortality, morbidity, and low remission, with abstinence rates of about 30% ten years after beginning care [21]. Over the past few years, the most significant increase in fatalities due to drug overdose was seen in cases involving illicitly-manufactured fentanyl and its analogues, with an average increase of 71% each year from 2013 to 2017 (whereas overdose deaths caused by prescription opioids and heroin have remained constant or declined, respectively) [22]. Notably, opioid-related mortality is highest among younger individuals (age 20 to 40 years) [23]. Additionally, prevalence of heroin use has more than doubled among TA adults in the past decade [24], with a significant increase in heroin overdose-related hospitalizations among 20–29 year olds in recent years [23]. The median age of onset for first heroin use, dependence, and treatment seeking for OUD patients is between 18–24 years of age, further underscoring the importance of emerging adulthood in OUD progression and treatment [25].

From 2012 to 2019, the number of TA adults receiving OUD treatment under New York State (NYS) Medicaid has increased more than 60%. Yet, TA adults often fall between two distinct treatment systems for SUD, most often placed in settings that are better tailored for adults, and where younger individuals feel out of place due to their unique social circumstances, such as having a shorter history of substance use and disorder [26], contributing to suboptimal treatment outcomes [27, 28].

Both the American Academy of Pediatrics and the American Society of Addiction Medicine recommend that medication be used in the treatment of young people with OUD [29–32]. Despite these recommendations, TA adults with OUD are less likely to receive MOUD than older adults [33–36]. In studies of young people with a diagnosis of OUD and covered by public and commercial insurance, one in four individuals received medications for OUD within six months of diagnosis [34, 35]. Only two randomized controlled trials of MOUD have been conducted specifically with TA adults and found that longer duration of MOUD (only 8–12 weeks in the trials) had improved outcomes over a shorter duration of MOUD [37, 38]. Studies generally find that longer retention in treatment is associated with improved outcomes [39–44], suggesting that stronger engagement in earlier treatment phases can lead to a better prognosis for TA adults [37, 38, 45–52].

Within SUD specialty treatment, MOUD increases treatment retention among TA adults [34, 53, 54]. However, TA adults generally have lower retention in specialty treatment than older adults [54, 55]. Even when MOUD is integrated into treatment, younger individuals are less adherent and more likely to leave treatment than older individuals [20, 27, 51, 56, 57]. Recent qualitative research highlights that TA adults acknowledge the life-saving potential of MOUD. Yet, they perceive receiving MOUD as stigmatizing and express ambivalence regarding MOUD, including fear of experiencing withdrawal symptoms, potential adverse effects, uncertainty regarding length of treatment, as well as concerns that MOUD might undermine self-sufficiency [58], which serves as a crucial marker of one’s transition to adulthood. There is a scarcity of studies of OUD treatment quality designed specifically for TA adults [59]. Observational studies of longitudinal cohorts are needed to identify if there are specific factors associated with use of MOUD, retention on MOUD, and adverse events related to treatment among TA adults [60].

Substance use and treatment for SUD occur within a broad social ecological context where the interplay of individual, community, and structural-level (including treatment program organizational) factors impact substance use and SUD treatment outcomes [61, 62]. Factors above the level of the individual, referred to as “social determinants of health,” such as structural racism and poverty, are potent fundamental causes of health inequalities [63].

TA adults have distinct individual-level characteristics that affect treatment engagement [4, 6, 64, 65]. They are more likely to have family members pressuring treatment [4, 64, 66, 67], and/or be involved with the criminal justice system [4, 26, 68, 69]. Criminal justice agencies and actors (e.g., judges and correctional staff) historically have been opposed to MOUD, preferring instead to rely on non-pharmacologic, non-evidence-based treatment approaches [70–73]. Furthermore, barriers to engagement, including lack of transportation or treatment programs that are high threshold settings, requiring frequent attendance at clinics and participation in highly structured protocols, challenge TA adults [60, 65, 67, 74–76]. TA adults are generally at ease interacting through modern telecommunications [6], with emerging shifts in telehealth that were prompted initially by the COVID-19 pandemic now offering new opportunities for treatment tailoring for this age group [77].

TA adults’ response to treatment also is affected by community-level factors that influence health and how they may engage with systems of care [78]. Variation in use of MOUD is associated with community social capital and local availability of programs offering MOUD [79]. MOUD is less available outside urban areas and often requires longer travel times that can be a barrier [74–76]. TA adults often contend with peers who use substances, local easy availability of substances, residential instability, hostile experiences with local law enforcement, and a lack of public transportation [80, 81]. Many TA adults from marginalized communities grapple with disparities in access and quality of care due to socioeconomic privation, racial/ethnic segregation and discrimination, and long-held community mistrust of medical and other institutions [67, 82–86].

Organizational-level factors also contribute to quality of care for TA adults [87]. Treatment programs that offer MOUD typically are larger organizations, hospital-affiliated, for-profit, and employ professional staff with advanced degrees (e.g., physicians, master’s level trained staff) [88, 89]. Data indicate that stigma of MOUD by staff in these healthcare settings has an impact on patients’ use of MOUD, even in programs that describe having a person-centered approach [90, 91]. Many specialty programs do not offer pharmacotherapy to youth [92], which has been associated with a lack of understanding of how to respond to the unique needs of these youth as they age into TA adults [93]. The impact of treatment program organization (e.g., program size and type), staffing (e.g., professional degrees), and clinical processes (e.g., use of telehealth, counseling modalities) on treatment quality practices and outcomes remains an area of ongoing exploration.

Considering the distinct circumstances and needs of TA adults, researchers and experts increasingly have called for additional research aimed at enhancing the quality of OUD treatment for this population [57, 60, 64]. Specific areas requiring investigation include strategies to improve MOUD access, engagement, and retention [94]. We extend prior studies by proposing a cohort-specific study that examines these questions using a social-ecological framework encompassing individual, community, and treatment program organization levels [18, 19, 34, 95].

## Materials and methods

### Conceptual framework

**Fig 1:**
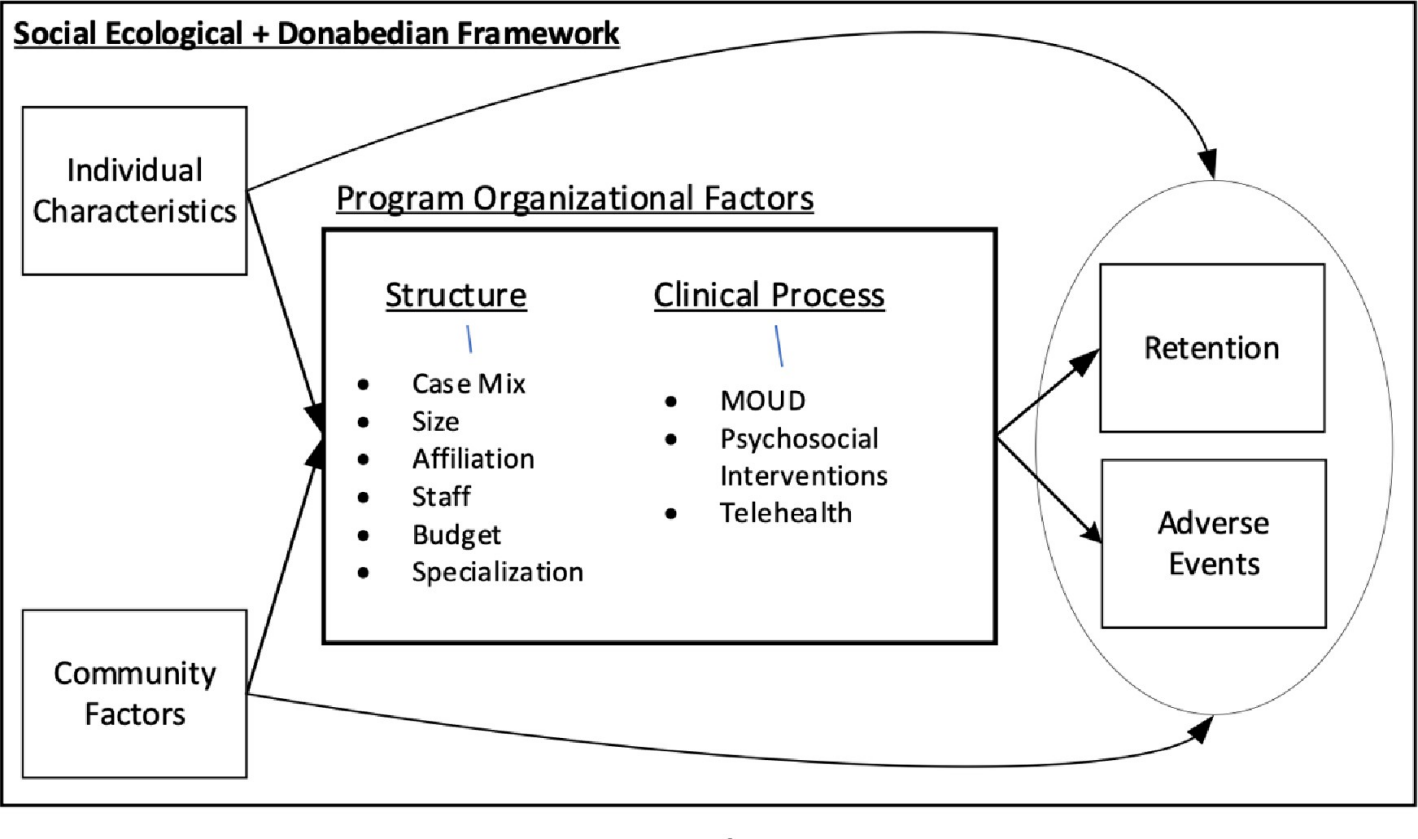
Social ecological and Donabedian conceptual framework.

As an interdisciplinary team of social scientists, clinical psychologists, and physicians, we recognize that the quality of treatment and care for SUD is structured by the interplay of individual, community-level, and treatment program organizational dynamics. As such, our study’s conceptual framework integrates key principles from the field of social ecology and the Donabedian healthcare quality framework [61, 62, 96–98], guiding our research questions and analysis. Fig 1 below depicts the conceptual framework. Primarily, we recognize the importance of considering individual characteristics (e.g., age, comorbidities) alongside the social and built environments. Second, we acknowledge that environments have a multi-level impact on treatment access and quality of care. For our study, we explore how program structural characteristics (e.g., program size and type) and clinical processes (e.g., use of telehealth; counseling modalities) structure outcomes, conditional on individual and community characteristics (e.g., socioeconomic status, racial/ethnic composition). Third, we incorporate a longitudinal perspective in our analysis to assess explicitly the evolving impact of individual, treatment program, and community-level factors on treatment quality practices and outcomes over several years. Fourth, we evaluate the quality of OUD-related treatment provided by organizations through the Donabedian framework, focusing on three key processes: 1) structural or institutional factors associated with capacity to provide care; 2) clinical protocols––namely processes––that guide interactions between staff and patients; and 3) outcomes, that is, whether patients achieve the goals for which they entered treatment [96–98].

### Study design and aims of the study

Our study introduces a retrospective, longitudinal cohort design to assess treatment quality practices and outcomes among TA patients with OUD in NYS using administrative data from 2010 through 2025. We will combine Medicaid claims and encounter data with data from the Client Data System (CDS), a state registry of treatment episodes in licensed SUD treatment programs. CDS is an Office of Addiction Services and Supports (OASAS) treatment registry that all licensed providers of SUD treatment in the state of New York use to enter admission and discharge data. Data include demographics (e.g., age, race/ethnicity, and marital status), level of functioning (e.g., housing status, health status, and comorbid mental health), criminal justice status, recent history and frequency of substance use, and recent SUD treatment history. This registry data will supplement information from the Medicaid data with additional socio-demographic and clinical information as well as SUD treatment that was not funded by Medicaid. Therefore, despite fragmentation in funding sources, the combination of these administrative data sets will yield a more inclusive record of treatment episodes. Through this targeted, innovative approach, we intend to identify the diverse treatment outcomes within this specific demographic across different program organizations and communities. The study has been reviewed by the Institutional Review Board of NYU Grossman School of Medicine (#s22-00914) and meets the criteria for exemption under 45 CFR 46.104(d). Study findings will be disseminated via national and international conferences, reports, and peer-reviewed publications.

#### Aim 1. Quantify the associations between MOUD use and individual, community, and program characteristics

Our first aim involves conducting an analysis of associations of MOUD use among TA adults and psychological and social determinants of health at the individual, community, and program organizational level. Using the CDS and Medicaid claims from NYS, we will assess the impact of demographic data (e.g., race/ethnicity, criminal justice involvement), housing status, comorbid mental health diagnoses, and community-level indicators (e.g., program availability, the social vulnerability index) on MOUD use. Additionally, we will examine the relationship between MOUD use and treatment program organizational characteristics, such as program size and type, telehealth volume, and integrated mental health treatment. We also will explore the influence of a range of factors on the use of different MOUD options (e.g., buprenorphine, methadone, or XR naltrexone) at individual, program, and community levels.

#### Aim 2. Assess program organizational characteristics associated with improved adherence and retention in OUD treatment, while accounting for individual and community factors

Our second aim focuses on identifying organizational factors that may be associated with improved medication adherence and retention in OUD treatment among TA adults. We will evaluate the impact of program structural factors (e.g., program size and type), program staff characteristics (e.g., professional degrees), and clinical process measures (e.g., use of telehealth, counseling modalities) on medication adherence and treatment retention, adjusting for individual and community-level characteristics.

#### Aim 3. Examine the association between program organizational characteristics and adverse OUD-related events (AE) such as overdose, while accounting for individual and community factors

Our third aim centers on investigating the nexus between program organizational characteristics and OUD-related adverse events among TA adults including SUD-related emergency department visits or hospitalizations, including for overdoses, and increases in levels of SUD care. We will examine the association between program structural factors (e.g., program size and type) and AE outcomes. Additionally, we will examine how program staff characteristics (e.g., professional degrees) impact AE. We also will explore the association between clinical care process measures (e.g., use of telehealth, counseling modalities) and AE outcomes.

### Sample

The study cohort will be drawn from combined Medicaid and CDS data and will include approximately 65,000 individuals ages 18 to 25 whose first treatment episode for OUD in Medicaid occurs in 2012 or later—that is, not preceded by a prior OUD-related treatment episode in 2010 or later. Individuals who are co-insured with Medicare (some people under 65 with certain disabilities or conditions) will be excluded because not all their healthcare events are represented in the Medicaid claims data. We estimate that approximately 5,000 individuals will meet these criteria each year. Our link rate among all Medicaid enrollees with OUD ages 18–25 is 93%. The demographic and clinical profile is expected to be generally stable across years. Each year we will select TA adults receiving their first treatment for OUD. Once someone has joined a cohort, that person is retained in the analytical dataset in all future years. By using the first treatment episode in Medicaid as our index episode for a young cohort, we are identifying individuals early in their OUD treatment continuum of care. We estimate that about 26,000 TA adults will enter treatment after the COVID-19 public health emergency related telehealth and other measures take effect.

### Analysis

The study uses rigorous methods to address questions about multilevel factors associated with MOUD use, retention, and adverse event mitigation. Table 1 below outlines measures aligned with the social ecology framework and data sources. Baseline patient, program, and community characteristics will be assessed at admission, while ongoing treatment and healthcare measures will be assessed monthly. We also will compare pre- and post-COVID-19 treatment patterns to examine care innovations on retention and outcomes, like telehealth and flexibility in methadone take-home doses. We present an outline of the analytical approach, specifying the strategies we intend to employ for each of our three aims.

**Table 1:** Summary of outcome and predictor variables and sources by level.

Concept	Variables	Source
***Individual Baseline Characteristics***		
Demographic	Client age; race/ethnicity; gender; education; housing status; employment; criminal justice involvement; SNAP; subsidized housing	OASAS Medicaid
Clinical complexity	Mental health conditions: serious mental health conditions (e.g., schizophrenia); PTSD; ADHD; chronic physical conditions; non-cancer pain conditions; HIV; HCV; opioid analgesic prescription	Medicaid
Substance Use Patterns	Age of onset; opioid use; other substances used (alcohol, stimulants (methamphetamine/cocaine/prescription stimulants); cannabis, synthetic cannabinoids); frequency of use; and injection drug use (IDU)	OASAS
Prior Healthcare Utilization	History of use of outpatient; inpatient; emergency department	Medicaid
***Individual-level Treatment Process***		
Psychosocial Interventions*	Behavioral treatment (i.e., group, individual, peer, or family counseling); telehealth; toxicology screening; treatment for mental health conditions; Medicaid reimbursed transportation; harm reduction supports; recovery center supports; youth clubhouse supports	Medicaid
Medication for OUD (MOUD)*	Use of medication for opioid use disorder (MOUD); MOUD option: methadone, buprenorphine, XR naltrexone; buprenorphine formulation and dose; change in dose from prior time period; days of supply	Medicaid
***Treatment Program Organizational Characteristics***		
Structure	Specialty opioid treatment program; program size (i.e., annual patient volume); % of TA adults among all clients; % OUD among clients; type (i.e., inpatient, outpatient); hospital system affiliation; integrated license for mental health services	OASAS Medicaid
Staffing	Number of staff; staff professionalization (e.g., degrees); gender and race/ethnicity; client-to-staff ratio	OASAS
Clinical Process	% MOUD (e.g., methadone, buprenorphine, XR naltrexone) among OUD clients; average monthly volume claims for individual, group, peer, and family counseling; average monthly volume claims for telehealth use; monthly volume for toxicology screening	Medicaid
***Community-level Characteristics***		
Access	Distances to the nearest Medicaid MOUD prescriber and other addiction treatment programs; low threshold buprenorphine programs; harm reduction providers or drug user health hubs providing MOUD services	Medicaid
Community Resources	Social vulnerability index (SVI) (i.e., composite measure of area-level vulnerability to adverse outcomes); racial/ethnic composition of community; population density; density of OUD among Medicaid members; proportion of Medicaid OUD members receiving MOUD	Census Medicaid
***Outcome Variables***		
MOUD Use*	Claim for methadone, buprenorphine (including formulation, e.g., sublingual tablets, sublingual film, subcutaneous injection, and implants), XR naltrexone	Medicaid
Adherence to MOUD*	Continued receipt of MOUD (continued methadone program appointments or renewal of buprenorphine or naltrexone prescriptions)	Medicaid
Retention*	Length of stay in treatment episode (in days)	Medicaid
Adverse Event*	SUD-related emergency department visit or hospitalization, including for overdose; increase in SUD level of care	Medicaid
Healthcare Utilization*	All-cause emergency department visits and hospitalizations	Medicaid

Table Notes: OASAS = CDS database; * Time-varying variables

#### Aim 1: Quantifying associations between MOUD use and social and ecological factors

To quantify the associations between MOUD use (e.g., methadone, buprenorphine, XR naltrexone) and individual, community, and treatment program organizational characteristics, we will use generalized linear mixed effects models (GLMM). The primary outcome of interest is whether an individual received MOUD during the treatment episode. We will include random effects to account for repeated measurements within individuals and clustering by program and county, and time fixed effects to address secular trends, such as the pre- and post-COVID-19 periods. We will test whether models can be simplified by replacing time fixed effects with time-related splines or power terms. We also will explore interactions among variables of substantive interest (e.g., varying the effect of criminal justice involvement on OUD treatment by community influences and program organizational factors). We will adopt a generalized estimating equations approach with robust standard errors to ensure the robustness of our findings. To address potential bias associated with correlations between predictors and random effects, we will utilize the Mundalk approach of mean-centering variables within county. A penalization approach (lasso regression) will be used for feature selection to address overfitting and collinearity. The validity of the approach will be ensured using cross-validation and sample splitting. Given the large sample size (>65,000) across multiple years, we are powered to detect conservative odds ratios (≥1.1) with 80% power at a two-sided alpha of 0.05 (assuming 10% overall prevalence of MOUD use). Higher prevalence will lead to improved power.

*Aim 1a*: *Associations with psychological and social determinants of health*. First, we will explore the association between MOUD use and psychological and social determinants of health at both the individual (e.g., housing status, comorbid mental health diagnoses) and community levels (e.g., social vulnerability index, program availability). For each group of measures, we will build distinct GLMMs. These models will be adjusted for age, sex, race, as well as other individual baseline and time-varying covariates (as specified in the measures table).

*Aim 1b*: *Associations with program organizational characteristics*. Second, we will focus on examining the connection between MOUD use and treatment program organizational characteristics (e.g., program size and type, volume of telehealth, integrated mental health treatment). Like Aim 1a, we will use GLMMs to analyze treatment program measures. These models will be adjusted for age, sex, race, other individual and community baseline measures, and other time-varying covariates (as detailed in the measures table). For aims 1a and 1b, estimates of odds ratios and their corresponding 95% confidence intervals will be reported.

*Aim 1c*: *Associations with MOUD options*. Third, we will investigate the factors linked to the use of different MOUD options (e.g., buprenorphine, methadone, or XR naltrexone) at individual, program, and community levels. To explore the association between medication option, provider proximity, and racial/ethnic patient characteristics, we will employ mixed effects multinomial logistic regression, while adjusting for individual, program, and county level covariates.

#### Aim 2: Evaluating program organizational factors influencing OUD treatment retention

This objective focuses on analyzing program organizational characteristics associated with improved medication adherence and retention in OUD treatment among TA adults, while controlling for individual and community characteristics related to retention in care. Employing time-to-event analysis, a set of statistical methodologies focusing on the time until an event occurs, we will focus on a) discontinuation of MOUD use following initiation during the treatment episode and b) treatment completion as our event outcome of interest. To accommodate the longitudinal structure of these data, we will employ extended Cox models. We will assess the validity of the proportional hazards (PH) assumption for time-invariant variables through log-log plots, observed versus expected plots, goodness-of-fit testing, and the use of time-dependent variables. Variables not adhering to the PH assumption will be assessed for interaction with time and, if necessary, incorporated into the final Cox model. We will assume non-informative censoring, meaning that censoring and the event risk are independent after conditioning on observable features.

These models will yield hazard ratios, critical in evaluating the hypotheses stated. To mitigate confounding, we will introduce individual, program, and community-level variables into the model, yielding adjusted hazard ratios alongside point estimates and 95% confidence intervals. The extensive confounding measures at individual, program, and community levels collectively address bias stemming from omitted or uncontrolled confounding variables. We will conduct robust sensitivity analyses and explore variation in association strength due to plausible instruments for program selection, such as patient travel distance to programs and concurrent MOUD use among other individuals in the same community. To prevent overfitting, we will apply techniques outlined in Aim 1. As a sensitivity analysis to consider the multilevel structure of the data, we will deploy extended mixed-effects Cox models. An extended Cox model can incorporate time-dependent factors [99]. A mixed-effects Cox model can incorporate multiple fixed and random effects [100]. Specifically, we will include a fixed effect for each individual, program, and community-level variable defined above and random effects for repeated measures of an individual over time, program, and community.

*Aim 2a*: *Program structural factors and adherence and retention in OUD treatment*. First, we will investigate the association between program structural factors (e.g., program size and type) and adherence and retention, guided by the hypothesis of adjusted hazard ratios being equal to 1.

*Aim 2b*: *Program staff characteristics and adherence and retention*. Second, we will examine the relationship between program staff characteristics (e.g., professional degrees) and adherence and retention, guided by the hypothesis of adjusted hazard ratios being equal to 1.

*Aim 2c*: *Clinical care process measures and adherence and retention*. Third, we will analyze the association between clinical care process measures (e.g., use of telehealth, counseling modalities) and adherence and retention, guided by the hypothesis of adjusted hazard ratios being equal to 1.

#### Aim 3: Examining program characteristics and adverse OUD-related events

Our final objective is to examine the association between treatment program organizational characteristics and adverse OUD-related events (AE) such as an SUD-related emergency department visit, including for overdose, while accounting for individual and community characteristics. Like Aim 2, we will employ time-to-event methods, focusing on the occurrence of the first adverse event. The adjusted hazard ratio of program structural factors, staff characteristics, and clinical care delivery features will be evaluated against a null hypothesis of 1. We will conduct sensitivity analyses akin to those described in Aim 2, ensuring the robustness of our results and conclusions. Applying the Lachin-Foulkes method to a study spanning 14 years, with variable drop-out rates, we determined that our sample size (>65,000) provides substantial power to detect clinically significant associations. Even under variable rates of attrition (10%-70%), we maintain adequate power to detect hazard ratios of 1.15 (or 0.87) with 80% power and a 5% type I error rate. Given the estimated study sample (>65,000), we are powered adequately to detect clinically meaningful associations.

*Aim 3a*: *Program structural factors and AE*. First, we will investigate the relationship between program structural elements (e.g., program size and type) and the occurrence of adverse OUD-related events (AE).

*Aim 3b*: *Program staff characteristics and AE*. Second, we will explore the connection between program staff attributes (e.g., professional degrees) and the incidence of adverse OUD-related events.

*Aim 3c*: *Clinical care process measures and AE*. Third, we will assess how clinical care processes (e.g., use of telehealth, counseling modalities) are associated with the prevalence of adverse OUD-related events.

## Discussion

Our study explores treatment quality outcomes for TA adults with OUD along three dimensions, encompassing the use of MOUD, treatment adherence and retention, and adverse event outcomes, such as overdose. We also will focus on the influence of organizational change and innovation on these outcomes, such as increased telehealth visits and flexibility in methadone take-home doses following onset of the COVID-19 federal public health emergency. By integrating large administrative datasets with insights from rigorous statistical analyses, our study will examine the socio-ecological factors influencing treatment quality and aid policy makers in establishing evidence-based standards for an understudied and vulnerable population.

With the number of TA adults seeking treatment for OUD on the rise, especially among those covered by Medicaid, this study has the potential to make an impact by improving the health outcomes of TA adults amidst the opioid crisis. One of our study’s strengths is that it is designed to study the quality of treatment for OUD that is specific to young adults. Adopting a socio-ecological perspective, we draw insights into how individual, program, and community-level characteristics impact how a specific population that is especially vulnerable to socio-ecological forces does over time. Given that OUD disproportionately affects individuals insured by Medicaid, the data linkage between the CDS and Medicaid claims is another key strength of the study.

We must acknowledge that our study has its limitations. The most critical limitation is that we will not be experimentally controlling for all factors related to program selection. Consequently, we will be tempered in making causal inferences about the associations we find [101, 102]. We address this limitation through the use of the rigorous statistical methods and sensitivity analyses described above. Studies of this type, which examine the impact of organizational quality in real-world settings, have a long tradition in informing both scientific practice and policy [102]. Another limitation of our study is that we do not compare the impact of multi-level factors on MOUD outcomes between TA and older adults, limiting our ability to discern if there are organizational factors that are more or less potently associated with MOUD outcomes for TA adults than for non-TA adults. Still, our study findings will yield important recommendations to engage and retain an underserved population in treatment at a pivotal life phase. Lastly, the administrative databases we use will not identify all aspects of care provision for OUD (e.g., staff decision-making and patient clinical experiences). Nevertheless, our study will be a valuable addition to the growing body of qualitative, ethnographic, and spatially oriented research that explores the impact of various mechanisms like social networks, institutional qualities, and structural vulnerability on substance-related risks and harms and treatment practices [103–105].

Over the past decade, there has been a significant increase in the prevalence of OUD among TA adults. Unfortunately, the existing OUD treatment framework inadequately serves this demographic, with lower rates of evidence-based treatment and increased early treatment attrition. Moreover, the heightened risk of substance-related adversities magnifies the issue. We need research to understand better the impact of existing types of care as well as inform policy makers, treatment providers, and the public about what individual, program, and community characteristics are associated with better quality of care. With the array of care options and the diversity inherent among TA adults, our study’s analytical methods will provide crucial information to address treatment gaps and disparities, benefiting this highly vulnerable age group.

## Status and timeline of the study

Access to both state treatment registry (CDS) and Medicaid claims has been acquired as of September 2023, but no data analysis has been done yet. Analysis is expected to start during the winter of 2023, after merging data from the two sources. Aim 1 analysis will begin during the winter of 2023, followed by Aims 2–3 concurrently. The study is set to be finished by the fall of 2025.
