# Supplementary material for: Lost in transition: A protocol for a retrospective, longitudinal cohort study for addressing challenges in opioid treatment for transition-age adults
Source: PLoS One. 2024 Aug 14;19(8):e0297567. doi: 10.1371/journal.pone.0297567 (PMC11324150; doi:10.1371/journal.pone.0297567)
Supplement: Peer review history

# Editor report: Decision Letter 0

**DOI:** 10.1371/journal.pone.0297567.r001

**Authors:** Jennifer Tucker

9 Jan 2024

Lost in transition: A protocol for a retrospective, longitudinal cohort study for addressing challenges in opioid treatment for transition-age adults

PONE-D-23-35728

Dear Dr. Neighbors,

We’re pleased to inform you that your manuscript has been judged scientifically suitable for publication and will be formally accepted for publication once it meets all outstanding technical requirements.

Within one week, you’ll receive an e-mail detailing the required amendments. Please modify your ethics statement to clarify that you were granted an exemption for the study, rather than approval as currently stated.  When these have been addressed, you’ll receive a formal acceptance letter and your manuscript will be scheduled for publication.

An invoice for payment will follow shortly after the formal acceptance. To ensure an efficient process, please log into Editorial Manager at http://www.editorialmanager.com/pone/, click the 'Update My Information' link at the top of the page, and double check that your user information is up-to-date. If you have any billing related questions, please contact our Author Billing department directly at authorbilling@plos.org.

If your institution or institutions have a press office, please notify them about your upcoming paper to help maximize its impact. If they’ll be preparing press materials, please inform our press team as soon as possible -- no later than 48 hours after receiving the formal acceptance. Your manuscript will remain under strict press embargo until 2 pm Eastern Time on the date of publication. For more information, please contact onepress@plos.org.

Kind regards,

Jennifer Tucker, PhD

Staff Editor

PLOS ONE

Journal requirements:

1. When submitting your revision, we need you to address these additional requirements.

Please ensure that your manuscript meets PLOS ONE's style requirements, including those for file naming. The PLOS ONE style templates can be found at 

https://journals.plos.org/plosone/s/file?id=wjVg/PLOSOne_formatting_sample_main_body.pdf and 

https://journals.plos.org/plosone/s/file?id=ba62/PLOSOne_formatting_sample_title_authors_affiliations.pdf

2. Please include your full ethics statement in the ‘Methods’ section of your manuscript file. In your statement, please include the full name of the IRB or ethics committee who approved or waived your study, as well as whether or not you obtained informed written or verbal consent. If consent was waived for your study, please include this information in your statement as well. 

Additional Editor Comments:

Prior to submission of the final version of your manuscript, please modify your ethics statement to clarify that you were issued an ethics exemption for the study.

eg. "The study has been reviewed by the Institutional Review Board of NYU Grossman School of Medicine (#s22-00914) and meets the criteria for exemption under 45 CFR 46.104(d). Study findings will be disseminated via national and international conferences, reports, and peer-reviewed publications."
